# Repositioning of 8-hydroxyquinoline derivatives as a new promising candidate for combating multidrug resistant *Neisseria gonorrhoeae*

**DOI:** 10.17179/excli2018-1602

**Published:** 2018-08-23

**Authors:** Ratana Lawung, Rungrot Cherdtrakulkiat, Sunanta Nabu, Supaluk Prachayasittikul, Chartchalerm Isarankura-Na-Ayudhya, Virapong Prachayasittikul

**Affiliations:** 1 Department of Clinical Microbiology and Applied Technology, Faculty of Medical Technology, Mahidol University, Bangkok 10700, Thailand; 2 Center of Data Mining and Biomedical Informatics, Faculty of Medical Technology, Mahidol University, Bangkok 10700, Thailand

**Keywords:** Neisseria gonorrhoeae, antimicrobial resistance, antibacterial activity, 8-hydroxyquinoline and derivatives

## Abstract

The multidrug resistance of *Neisseria gonorrhoeae* becomes a public health problem worldwide, especially the strain H041 that showed the decrease susceptibility to ceftriaxone which is the last resort for gonorrhea treatment. Therefore, the simultaneous discovery and development of a new compound to fight this pathogen is urgently required. In this study, 8-hydroxyquinoline (8HQ) and derivatives were evaluated for their antimicrobial activities against the gonococcal pathogen using spectinomycin as the reference drug. The results showed that 8HQ derivatives gave an excellent antimicrobial potency. Particularly, the dihalogenated 8HQ (iodoquinol, clioquinol and 5,7-diCl-8HQ) exerted the high activity with MIC range of 0.08-0.15 μM, 0.10-0.20 μM and 0.28-0.56 µM, respectively, compared with the reference drug (MIC = 16 μg/mL or 48.14 μM). Moreover, these compounds were also shown to be non-cytotoxic/very high safety index. The findings reveal that these three compounds could be further developed as a new antimicrobial agent for fighting the gonorrheal disease.

## Introduction

The first *Neisseria gonorrhoeae* strain H041 has been discovered as a superbug since 2011. It is highly resistant to extended-spectrum cephalosporin (ESC), ceftriaxone, that is the last resort for gonorrhoea treatment (Ohnishi et al., 2011[12]). In the past, the *N. gonorrhoeae* has been continuingly reported as the multidrug resistances starting from sulfonamides, penicillin, tetracycline, spectinomycin, ciprofloxacin and azithromycin, respectively (Unemo and Shafer, 2014[17]). Nowadays, the emerging of gonococcal isolates with decrease susceptibility to ceftriaxone and cefixime, the first-line drugs for treatment, has been reported. Even though the new antimicrobials have been proceeding in the clinical trial, but the superbug has adapted itself to resist in a few years. Therefore, it is an urgent issue to discover new antimicrobial agents to fight this pathogen. Recently, the drug repurposing strategy (Corsello et al., 2017[9]) has been recognized for this purpose. The approach gives the possible usage of the approved drugs or investigated drugs/compounds for new therapeutic treatments. This can reduce the expensive and time consuming processes that are required for new drug candidates (Anighoro et al., 2014[1]).

8-Hydroxyquinoline (8HQ) and derivatives have been documented and used as antimicrobial, antimalarial, antiparasitic, anticancer as well as antineurodegenerative and antioxidant agents (Chan-on et al., 2015[4]; Oliveri and Vecchio, 2016[13]; Prachayasittikul et al., 2013[14]; Song et al., 2015[15]). Particularly, nitroxoline and clioquinol (8HQ derivatives) have been reported as antibacterials used in urinary tract infection, and as antiamoebic in gastrointestinal tract infection, respectively (Oliveri and Vecchio, 2016[13]; Wagenlehner et al., 2014[18]). 8HQ and derivatives have been found to exhibit potent antimicrobial activity by our group (Cherdtrakulkiat et al., 2016[5]). To pursue the study, therefore, the 8HQ and derivatives (**1**-**7**) have been evaluated for antimicrobial activity against *N. gonorrhoeae*. Interestingly, the diiodo derivative of 8HQ (iodoquinol) can exert the excellent potency against this pathogen, including the multidrug resistant strains.

## Materials and Methods

### Tested compounds

**Figure 1:**
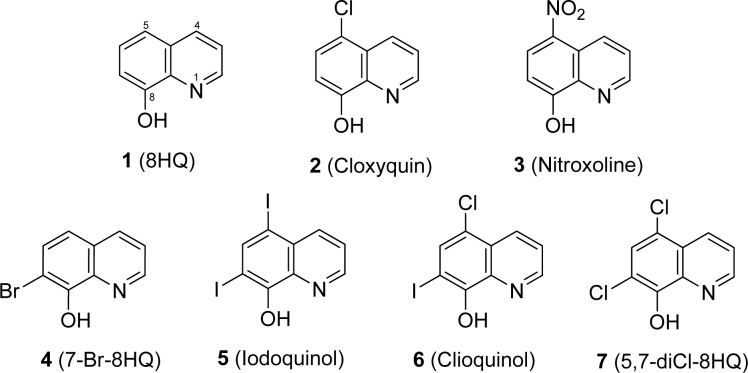
Chemical structures of 8HQ and its derivatives (1-7)

Tested compounds (i.e. 8HQ and derivatives) and antibiotics are comercially available. 8-Hydroxyquinoline (8HQ, **1**); 5-chloro-8-hydroxyquinoline (5-Cl-8HQ or cloxyquin, **2**); 5-nitro-8-hydroxyquinoline (5-NO_2_-8HQ or nitroxoline, **3)**; 7-bromo-8-hydroxyquinoline (7-Br-8HQ, **4**); 5,7-diiodo-8-hydroxyquinoline (5,7-diI-8HQ or iodoquinol, **5**) and 5-chloro-7-iodo-8-hydroxy- quinoline (5-Cl-7-I-8HQ or clioquinol, **6**) were obtained from Sigma-Aldrich. 5,7-Dichloro-8-hydroxyquinoline (5,7-diCl-8HQ, **7**) was purchased from Acros Organics. Spectinomycin was supplied with Bio Basic Inc. Chemical structures of the tested compounds are shown in Figure 1(Fig. 1). 

### Bacterial selection, culture conditions and storage

All *N. gonorrhoeae* isolates were kindly provided by the National Center of Sexually Transmitted Diseases, Bangrak Hospital, Bangkok, Thailand. A quality control reference strains: ATCC 49226 and WHO reference strains: WHO K, WHO L, WHO O and WHO P were used as control strains (Unemo et al., 2009[16]). *N. gonorrhoeae* strain displaying resistance to extended spectrum cephalosporins (ceftriaxone MIC = 1 µg/mL and cefixime MIC = 4 µg/mL) was also included while all clinical isolates were collected in years 2014-2015. Antimicrobial resistance phenotypes of penicillin (PEN), tetracycline (TET), ciprofloxacin (CIP), ceftriaxone (CRO), cefixime (CFM), spectinomycin (SPE), azithromycin (AZM) and gentamycin (GEN) were determined by disk diffusion and E-test methods (Nachnani et al., 1992[11]). Production of β-lactamase enzyme was detected using nitrocefin disks. The susceptibility interpretive criteria followed the Clinical and Laboratory Standards Institute (CLSI) documents (CLSI, 2014[8]) for all drugs, except for azithromycin and gentamicin that followed the Centers for Disease Control and Prevention guideline (CDC, 2012[2]) and Chisholm et al. (2011[6]), respectively*.* Total of 34 isolates were selected based on a variety of antimicrobial resistant phenotypes (Table 1(Tab. 1)).* N. gonorrhoeae* isolates were grown on chocolate agar, and incubated at 36 ± 1 °C with 5 % CO_2 _in a humidified environment for 20 to 24 hours. All isolates were stored at -80 °C in skimmed milk containing 10 % (v/v) glycerol. 

### Antimicrobial susceptibility testing

Antimicrobial activity of the tested compounds was determined by an agar dilution method according to CLSI guidelines (CLSI, 2012[7]), and *N. gonorrhoeae* ATCC 49226 was used as a standard strain. The tested compounds were dissolved in dimethyl sulfoxide (DMSO), in which the DMSO concentration did not exceed 1 % of the total agar volume. Gonococci (GC) agar plates, supplemented with 1 % define growth supplement containing 2-fold dilution of the tested compounds, were prepared at a final concentration ranging from 0.01 to 128 µg/mL. Spectinomycin was used as a control antibiotic with a final concentration range of 4-128 µg/mL. GC agar supplemented with 1 % define growth supplement, and GC agar supplemented with 1 % define growth supplement containing 1 % DMSO were used as controls.

Colonies of *N. gonorrhoeae* from overnight growth on chocolate agar were directly suspended in Muller Hinton broth (Difco BD Biosciences, Canada), and were adjusted to a density equivalent to a 0.5 McFarland standard. Bacterial suspensions were inoculated onto control plates and tested compound plates (as the above preparation), and incubated at 36 ± 1 °C with 5 % CO_2 _in a humid environment. The antimicrobial susceptibility results were recorded after 20 to 24 hours of the incubation. The minimum inhibitory concentration (MIC) was determined as the lowest concentration that showed complete growth inhibition. 

## Results

### Antimicrobial activity

8HQ and derivatives (**1**-**7**) were evaluated for the antimicrobial activity against 6 reference strains and 34 clinical isolates of *N. gonorrhoeae* using the agar dilution method (Table 1(Tab. 1)). *N. gonorrhoeae* ATCC 49226 was used as a quality control strain and tested with SPE, the reference drug. MIC value of the SPE was shown to be 16 µg/mL (48.14 µM) as recommended by the CLSI (8-32 µg/mL). Moreover, the WHO reference strains of *N. gonorrhoeae* showed the MIC value for SPE as observed for the standard strain (16 µg/mL), except for the WHO O strain (>1,024 µg/mL). The parent compound 8HQ (**1**) showed the MIC range of 27.56-55.11 µM (4-8 µg/mL) against all the clinical isolates. The dihalogenated compounds such as iodoquinol (**5**) and the clioquinol (**6**), exerted the highest antimicrobial activity with MICs range of 0.08-0.20 µM (0.03-0.06 µg/mL) and followed by the 5,7-diCl-8HQ (**7**, MIC = 0.28-0.56 µM or 0.06-0.12 µg/mL). Among the monohalogenated 8HQs, cloxyquin (**2**) and 7-Br-8HQ (**4**) showed the comparable MIC range (2.23-5.57 µM) as noted for the nitro compound (nitroxoline **3**, 2.63-5.26 µM). Notably, the diiodo groups at positions-5 and -7 of 8HQ (**5**) exhibited more potent antimicrobial activity against *N. gonorrhoeae* than the dichloro derivative (**7**). Moreover, the dihalogenated 8HQ (**5**-**7**) displayed higher antimicrobial activity compared with the monohalogenated and nitro compounds (**2**-**4**). 

Interestingly, the mono-drug resistant (TET^R^, TRNG and CIP^R^) of gonococcal isolates had the MIC range that are not different from 2-fold of the MIC values of double-drug resistant and multi-drug resistant isolates (Table 1(Tab. 1)). Moreover, the TRNG (high-level of tetracycline resistant) which is the plasmid-mediated resistance, and the TET^R^ (chromosomally-mediated resistance) also showed the same range of MIC values, for example, 8HQ (**1**) showed the MIC of 55.11 µM against both TRNG and TET^R^ isolates. Apparently, the 8HQ derivatives (**2**-**7**) exerted the great antimicrobial activity to all *N. gonorrhoeae* isolates including the high resistant isolates (CRO^R^ and CFM^R^). Although the parent compound (8HQ) showed the lowest antimicrobial activity, its MIC range (27.56-55.11 µM) is not different from the reference drug (SPE; MIC = 24.07-3,622.65 µM). Therefore, all of the tested compounds should be selected for further development as a potential drug against this pathogen.

### Selectivity index 

Selectivity index (SI) of the 8HQ and derivatives (**1**-**7**) is demonstrated in Table 2(Tab. 2) based on the cytotoxicity of normal MRC-5 cell lines (Cherdtrakulkiat et al., 2016[5]), in which the SI = IC_50_ of MRC-5 cell line/ MIC range of *N. gonorrhoeae* isolates. The results (Table 2(Tab. 2)) showed that the iodoquinol (**5**) had the highest range of SI values (>1,574.5 to >839.73). Moreover, the clioquinol (**6**) also gave the high SI value (761.70-380.85). Therefore, compounds** 5** and **6** are the most potent compounds with high safety index that could be applied as an antimicrobial drug against *N. gonorrhoeae*. Among the halogenated 8HQ, all dihalogenated 8HQ (**5**-**7**) displayed the higher SI values (>1,574.5 to 49.96) whereas monohalogenated 8HQs showed the lower SI (29.40 to 4.95), especially as noted for 7-Br-8-HQ (**4**, SI = 9.91-4.95). On the other hand, the parent compound (8HQ) was the only one that provided the lowest SI value (0.23-0.11).

## Discussion

8HQ and derivatives have a variety of multi-functional treatments as antibacterial, antiparasitic, antifungal and antimalarial agents (Prachayasittikul et al., 2013[14]; Song et al., 2015[15]). In particular, nitroxoline (**3**) has been used as an anti-neurodegenerative drug for Alzheimer disease (Jiang et al., 2011[10]). In this study, seven 8HQ and derivatives have shown high antimicrobial potency against all the clinical isolates of *N. gonorrhoeae* including the reference strains from WHO, when compared with SPE, the reference drug. The dihalogenated 8HQ (**5**-**7**) exerted the excellent antimicrobial potency (MIC range of 0.08-0.56 µM). Especially, the compound that has diiodo groups (**5**, MIC = 0.08-0.15 µM) showed higher potency than the dichloro groups (**7**), in which **5** > **6** > **7**.

In the previous study (Cherdtrakulkiat et al., 2016[5]), iodoquinol (**5**) displayed weaker antimicrobial activity (MIC ≥644.92 µM) against most of the bacterial strains (gram positive and gram negative bacteria, and diploid fungi and yeast). The iodoquinol drug (**5**) was reported to exhibit antiparasitic activity against *Dientamoeba fragilis* ATCC 30948 with the minimal amoebicidal concentration of 128 μg/mL (Chan et al., 1994[3]). However, monohalogenated derivatives of 8HQ (**2** and** 4**) also displayed the antimicrobial activity with the MIC range of 2.23-5.57 µM. Interestingly, the MICs of 8HQ and derivatives were shown to be almost equivalent to the MIC values among the mono-drug resistant, double-drug resistant and the multi-drug resistant *N. gonorrhoeae* including the high resistant strain (CRO^R^ and CFM^R^).

**Figure 2:**
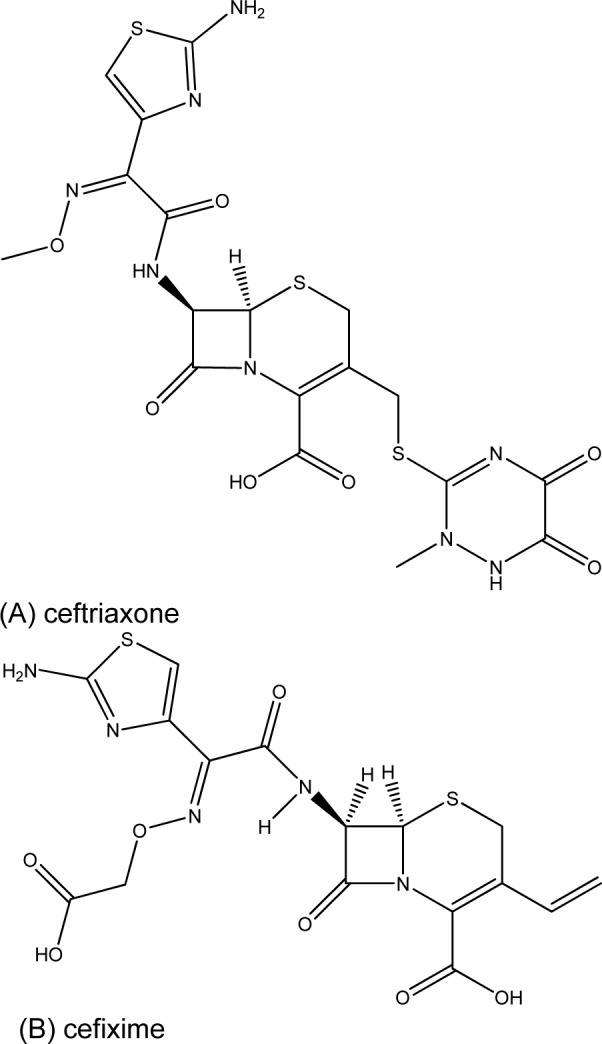
Chemical structures of the first-line drugs

The striking active compound **5,** bearing 8HQ privileged structure, is a small molecule compared with the complex chemical structure of cephalosporin (ESC) namely ceftriaxone and cefixime (Figure 2(Fig. 2)), which are the first-line drugs for gonococcal treatment. In addition, the diiodo compound **5** has shown to be non-cytotoxic (Cherdtrakulkiat et al., 2016[5]) to the normal cell line (MRC-5, IC_50_ >125.96 µM) where- as the parent 8HQ (**1**) displayed high cytotoxicity with the IC_50_ value of 6.27 µM (Table 2(Tab. 2)). Importantly, it should bear in mind that this is the drug repurposing strategy to make benefit from the existing compounds/drugs for combating the gonorrheal disease.

Owing to non-cytotoxicity and or high safety index, the 8HQ derivatives (**5**-**7**) with the great antimicrobial property should be selected as a new alternative compound that could be further developed as a gonorrheal treatment drug in the future. 

In conclusion, the findings reveal the advantage of dihalogenated 8HQs as new drug development in gonorrheal therapy, and as a combination drug with conventional treatments.

## Acknowledgement

This work is supported by Center of Excellence on Medical Biotechnology (CEMB), S&T Postgraduate Education and Research Development Office (PERDO), Office of Higher Education Commission (OHEC), Thailand, and Annual Government Grant under Mahidol University (2557-2559 B.E.), Thailand.

## Figures and Tables

**Table 1:**
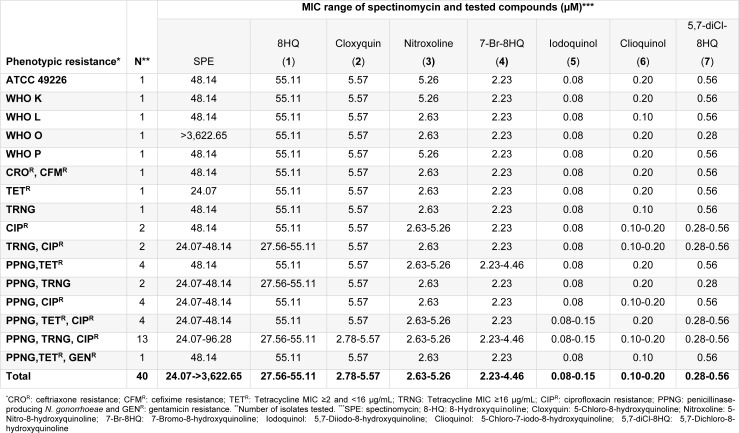
MIC values of spectinomycin and compounds 1-7 against multidrug resistant *N. gonorrhoeae*

**Table 2:**
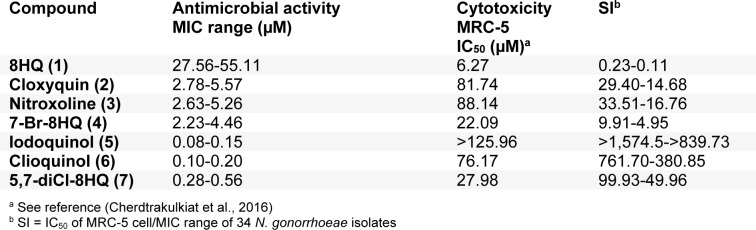
Selectivity index (SI) of 8-HQ and derivatives (1-7)
